# Muscular Dystrophy Muscle Preservation: A Comparative Systematic Review of Exercise and Cannabidiol (CBD) Interventions

**Published:** 2026-03-26

**Authors:** Andre Aabedi, Daniel Masiach, Devendra K. Agrawal

**Affiliations:** Department of Translational Research, College of Osteopathic Medicine of the Pacific, Western University of Health Sciences, Pomona, California 91766 USA

**Keywords:** Anti-oxidation, Cannabidiol, Exercise therapy, Fibrosis, Inflammation, Muscle mass preservation, Muscular dystrophy, Neuromuscular disease, Non-pharmacologic interventions

## Abstract

Muscular dystrophy comprises a heterogeneous group of inherited neuromuscular disorders characterized by progressive muscle degeneration, weakness, and functional decline. In the absence of curative pharmacologic therapies, non-pharmacologic strategies aimed at preserving muscle mass and function are of growing clinical importance. Exercise therapy is an established cornerstone of conservative management, whereas cannabidiol supplementation has emerged as a novel experimental intervention supported primarily by preclinical evidence. This systematic review synthesizes and compares the available evidence on exercise therapy and cannabidiol supplementation with respect to muscle mass preservation in muscular dystrophy. Exercise interventions, including aerobic, resistance, and functional training, demonstrate modest but consistent benefits in functional performance, endurance, and quality of life, with acceptable safety profiles when appropriately individualized. In contrast, cannabidiol supplementation has shown promising anti-inflammatory, anti-fibrotic, and myoprotective effects in dystrophic animal models, yet lacks robust human clinical data. Comparative analysis suggests overlapping anti-inflammatory and antioxidant mechanisms, raising the possibility of synergistic benefit; however, combined intervention strategies remain untested. Overall, exercise therapy remains the most evidence-supported approach for muscle preservation in muscular dystrophy, while cannabidiol supplementation warrants cautious investigation through rigorously designed clinical trials. Future research should prioritize head-to-head randomized controlled trials, standardized outcome measures, and subtype-specific therapeutic strategies to clarify the independent and complementary roles of these interventions.

## Introduction

Muscular dystrophy (MD) encompasses a heterogeneous group of inherited neuromuscular conditions marked by progressive skeletal muscle degeneration and weakness. The pathophysiology generally stems from mutations in genes coding for proteins critical to muscle fiber integrity, dystrophin mutations in Duchenne muscular dystrophy (DMD) being the most well-characterized example. Loss of dystrophin compromises sarcolemmal stability and triggers a cascade of downstream events including dysregulated calcium handling, oxidative stress, persistent inflammation, and defective muscle regeneration. These processes converge to drive irreversible muscle atrophy and functional deterioration [1].

Because no curative pharmacologic treatments currently exist, attention has shifted toward non-pharmacologic interventions that might slow muscle loss and enhance quality of life. Exercise therapy has become a mainstay of conservative management. Accumulating evidence indicates that carefully designed strength and endurance training programs can enhance muscle function, improve balance, and reduce perceived exertion across various MD subtypes, with acceptable safety profiles [2-5]. Nevertheless, questions persist regarding optimal exercise modalities and intensities, and current practice favors individualized programming to balance therapeutic benefit against potential harm [3, 6, 7].

More recently, cannabidiol (CBD) supplementation has attracted interest as a potential adjunct therapy, largely due to its anti-inflammatory and cytoprotective effects. Studies in dystrophic mouse models have shown that CBD reduces muscle inflammation, restores autophagic flux, and supports myotube formation, with corresponding improvements in muscle histology and myofiber diameter [8-10]. While these preclinical findings are encouraging, human clinical data remain scarce, and questions about long-term safety have yet to be fully addressed.

This review systematically compares the evidence supporting muscle mass preservation with exercise therapy versus cannabidiol supplementation in muscular dystrophy. By integrating findings from both intervention types, we seek to clarify their individual contributions and explore whether they might work synergistically in managing progressive muscle loss in MD.

### Exercise Therapy in Muscular Dystrophy

Recent clinical studies and systematic reviews have expanded upon existing guideline recommendations by elucidating potential mechanisms underlying exercise benefits in muscular dystrophy. Accumulating data suggest that exercise may improve mitochondrial efficiency and neuromuscular activation while exerting anti-inflammatory effects, possibly through AMPK-dependent mitochondrial biogenesis and mitophagy induction [11]. Studies using dystrophin-deficient animal models have shown that high-intensity interval training combined with isometric contractions can improve fatigue resistance and restore aspects of mitochondrial function, lending mechanistic support to exercise-based interventions [11].

The exercise modalities investigated span aerobic training, resistance training, low-load blood flow restriction, and functional training programs [4],[12]. Aerobic activities such as cycling and swimming have yielded modest gains in endurance and walking distance, though their impact on muscle strength appears limited. Supervised resistance training programs, when appropriately tailored, have been associated with increased knee flexor strength and improvements in functional tasks including sit-to-stand transitions and stair climbing in ambulatory adults with limb-girdle, Becker, and facioscapulohumeral dystrophies [13]. Functional and multicomponent exercise regimens have similarly shown promise for improving balance, gait parameters, and perceived exertion, particularly in individuals with myotonic dystrophy and limb-girdle muscular dystrophy [2],[14].

While functional and endurance outcomes are generally favorable, improvements in muscle mass and strength tend to be modest and inconsistent across studies. Meta-analytic data indicate that exercise can extend walking endurance by approximately 17 meters on average, yet fails to produce significant strength gains in facioscapulohumeral or myotonic dystrophy populations [4]. Resistance training may confer small benefits to specific muscle groups and functional performance measures, whereas moderate aerobic exercise has been linked to enhanced respiratory and cardiac function along with increased serum adiponectin, a marker potentially reflective of therapeutic benefit [12-13]. The overall quality of evidence, however, remains limited by small sample sizes and methodological heterogeneity across trials [15].

Safety remains a paramount concern. Most published studies describe exercise interventions as well tolerated, with adverse events typically confined to mild muscle soreness or joint discomfort [5], [15]. High-intensity and eccentric exercise protocols are generally discouraged given the heightened risk of muscle damage, and individualization of training parameters is essential to prevent excessive fatigue and pain [14], [16]. Barriers to sustained adherence include the requirement for professional supervision, potential for overexertion, and psychosocial factors such as depression and diminished self-esteem that may hinder participation. Consequently, personalized and multidisciplinary management strategies, including early attention to respiratory complications, are increasingly advocated to maximize both safety and long-term engagement [3], [5].

### Cannabidiol Supplementation in Muscular Dystrophy

Cannabidiol supplementation has garnered attention in muscular dystrophy research based on preclinical evidence suggesting anti-inflammatory, anti-fibrotic, analgesic, and endocannabinoid-modulating properties. However, human clinical data remain exceedingly sparse and heterogeneous.

The proposed mechanisms of CBD action involve reduction of macrophage infiltration and pro-inflammatory cytokine production, alongside anti-fibrotic effects that diminish excessive collagen deposition in dystrophic muscle tissue. Studies utilizing mdx mouse models of Duchenne muscular dystrophy have demonstrated that CBD and structurally related cannabinoids can attenuate muscle inflammation, fibrosis, and necrosis while simultaneously improving myofiber size and contractile function. These therapeutic effects appear partially mediated through modulation of transient receptor potential (TRP) channels and the broader endocannabinoid system, including CB1 and CB2 receptors, which have been implicated in both muscle regeneration and immunomodulation [8-10], [17]. Additionally, CBD exhibits analgesic properties that may prove relevant for pain management in neuromuscular conditions [18].

Preclinical investigations in mdx mice have shown that full-spectrum CBD oil administered at 10 mg/kg/day for 14 days improved multiple histopathological parameters, including reductions in centrally nucleated fibers, IgG-positive myofibers, inflammatory and fibrotic areas, and serum creatine kinase levels, all without apparent toxicity at this dosage [10]. Higher doses of CBD or cannabidivarin (CBDV), up to 60 mg/kg, have similarly preserved locomotor activity and restored autophagic processes in dystrophic models [9].

Despite these encouraging preclinical findings, no clinical trials evaluating CBD specifically in muscular dystrophy populations have been published to date. Available safety and pharmacokinetic data are derived primarily from studies in other conditions, notably epilepsy, where pharmaceutical-grade oral CBD (Epidiolex) has generally been well tolerated but associated with hepatotoxicity, gastrointestinal symptoms, and clinically significant drug interactions [18-19]. The dosing regimens employed in animal studies vary considerably, and appropriate human dosing for muscular dystrophy applications has not been established.

Several critical gaps hinder clinical translation. Substantial heterogeneity exists across CBD formulations, ranging from full-spectrum botanical extracts to purified isolates, with corresponding variability in bioavailability and pharmacologic activity. Standardized dosing protocols are lacking, and the absence of controlled trials in neuromuscular disease populations leaves efficacy and safety profiles inadequately characterized [20]. Furthermore, concerns regarding potential hepatotoxicity, drug-drug interactions, and unknown long-term effects in individuals with neuromuscular disorders warrant careful consideration. Patient adherence may be further compromised by inconsistent product composition, cost barriers, and insufficient regulatory oversight of non-pharmaceutical CBD preparations [18, 20].

### Comparative Synthesis

Exercise therapy represents an established intervention for mitigating muscle loss in muscular dystrophy, whereas cannabidiol supplementation constitutes an emerging experimental approach supported by encouraging preclinical findings but minimal clinical validation.

The evidentiary basis for these interventions differs markedly in strength and maturity. Exercise therapy is substantiated by numerous systematic reviews and meta-analyses documenting improvements in functional performance, endurance capacity, and perceived exertion, alongside generally acceptable safety profiles in muscular dystrophy cohorts [2-4, 15]. Conversely, the therapeutic potential of CBD rests predominantly on animal model data and preclinical investigations that have demonstrated histopathological improvements, attenuated inflammatory responses, and increased myofiber diameter in dystrophic mice, with human clinical evidence remaining conspicuously absent [8, 9, 18].

Both modalities appear to share certain mechanistic features, particularly their anti-inflammatory and antioxidant properties. Exercise has been shown to reduce muscle oxidative stress, inflammation, and fibrotic remodeling while promoting regenerative capacity and hypertrophic adaptation [21]. CBD similarly suppresses inflammatory mediators including IL-6, NF-κB, and TNF-α, reduces oxidative stress biomarkers, and upregulates cytoprotective pathways such as Nrf2 and HO-1, collectively contributing to muscle preservation [9].

However, these interventions also exhibit distinct therapeutic profiles. Exercise therapy confers its primary benefits through enhanced functional capacity, improved endurance, and better quality of life, though its effects on absolute muscle strength remain modest. CBD may offer unique advantages through reduction of fibrotic tissue deposition, pain modulation, and promotion of muscle regeneration mediated by autophagic flux and TRP channel activation, alongside direct anti-fibrotic and analgesic actions [22].

The possibility of synergistic benefit warrants consideration. Exercise could facilitate muscle adaptation and functional gains, while CBD might attenuate exercise-induced muscle damage and inflammatory responses, theoretically permitting greater exercise tolerance and accelerated recovery. Nevertheless, combined intervention protocols have not undergone systematic investigation in muscular dystrophy populations, and neither optimal dosing strategies nor timing parameters have been defined [6, 22].

Safety profiles and adherence considerations differ between approaches. Exercise therapy demonstrates acceptable tolerability when appropriately individualized and supervised, although sustained participation may be hindered by fatigue, pain, and progressive functional decline [23]. CBD appears reasonably safe in preclinical models and select clinical musculoskeletal applications, yet dose-dependent cytotoxicity has been documented in vitro, and long-term safety data in neuromuscular disease populations are unavailable [10, 18]. Regulatory considerations further complicate clinical application, as CBD lacks approval for muscular dystrophy indications and carries potential for pharmacokinetic interactions with concomitant medications [19].

In summary, exercise therapy remains the primary evidence-based strategy for muscle preservation in muscular dystrophy, with substantial support for functional benefits and safety. CBD supplementation shows promise but requires rigorous clinical validation before definitive recommendations can be made. Although their convergent anti-inflammatory and antioxidant mechanisms raise the intriguing prospect of synergistic efficacy, combined treatment protocols should be pursued cautiously pending availability of empirical data demonstrating safety and benefit.

### Limitations of the Evidence

Exercise therapy presently enjoys more consistent empirical support for muscle preservation in muscular dystrophy than cannabidiol supplementation, though both therapeutic approaches remain constrained by small sample sizes, substantial disease heterogeneity, and absence of standardized outcome measures.

The evidence base for exercise therapy is anchored in multiple systematic reviews and meta-analyses indicating that structured programs, particularly those combining aerobic and resistance training components, can yield improvements in muscle strength, hypertrophy, and functional performance across various muscular dystrophy subtypes including facioscapulohumeral (FSHD), limb-girdle (LGMD), and Duchenne muscular dystrophy. Illustratively, a 24-week combined exercise intervention in FSHD patients resulted in increased muscle satellite cell abundance and induced myofiber hypertrophy without accelerating degenerative processes or telomere attrition, suggesting maintenance of regenerative potential [24]. Nonetheless, the aggregate quality of evidence remains low to very low, with most investigations characterized by limited sample sizes, heterogeneous training protocols, and inconsistent operational definitions of muscle mass encompassing measures such as muscle strength, fiber diameter, and functional testing [2], [4], [15]. Exercise prescription requires careful individualization with respect to intensity and modality, as poorly designed protocols may precipitate harm or yield minimal benefit, underscoring the need for disease-specific programming guidelines [5], [14].

Regarding cannabidiol supplementation, preclinical investigations utilizing mdx mouse models of DMD have demonstrated that CBD and full-spectrum CBD oil preparations can attenuate inflammatory responses, restore autophagic function, and improve histopathological indices of muscle health, including augmented myofiber cross-sectional area alongside reduced fibrosis and inflammatory cell infiltration [9-10]. Human clinical data, however, remain exceedingly sparse, with no adequately powered trials examining CBD effects on muscle mass in muscular dystrophy populations. Dosing strategies in animal studies exhibit considerable variability, ranging from 10 to 60 mg/kg/day, and consensus regarding standardized outcomes and human safety parameters has not been achieved. Moreover, the sole FDA-approved CBD formulation (Epidiolex) carries no indication for neuromuscular disease, rendering its application in this context strictly experimental [8, 19].

Both interventions confront substantial methodological obstacles, including limited sample sizes, phenotypic heterogeneity across muscular dystrophy subtypes, lack of consensus on outcome measures for muscle mass assessment, and considerable protocol variability. CBD research faces the compounding challenge of minimal human trial data and inconsistent dosing regimens, substantially complicating direct comparisons with exercise-based interventions. While exercise studies are more numerous, they nevertheless exhibit persistent methodological weaknesses and inconsistent reporting practices that constrain definitive conclusions.

In summary, exercise therapy benefits from a substantially larger body of human clinical evidence and has been integrated into routine care recommendations for muscular dystrophy, although questions regarding optimal training protocols remain unresolved. CBD demonstrates encouraging effects in preclinical animal models but currently lacks adequate human evidence to support its use for muscle mass preservation in muscular dystrophy populations. Both interventions require rigorous, well-designed clinical trials with standardized methodologies and sufficient sample sizes to definitively establish their therapeutic roles and inform evidence-based practice guidelines [2, 3, 15, 24].

### Future Directions

Future investigations comparing exercise therapy and cannabidiol supplementation for muscle preservation in muscular dystrophy should prioritize several key areas: rigorously designed head-to-head randomized controlled trials (RCTs) including combination therapy arms, development and validation of sensitive biomarkers, disease subtype-specific exercise prescriptions, and dose-finding studies utilizing pharmaceutical-grade CBD formulations.

The field currently lacks adequately powered, methodologically sound RCTs that directly compare exercise therapy, CBD supplementation, and their combined use in muscular dystrophy populations. While preclinical evidence suggests CBD may ameliorate muscle pathology in dystrophic animal models, and systematic reviews support the safety and modest efficacy of exercise interventions, these modalities have been studied largely in isolation [2-4], [9], [10], [25]. Head-to-head comparative trials incorporating combination therapy arms are essential to elucidate potential additive or synergistic effects and inform evidence-based clinical practice guidelines.

Future studies should integrate sensitive, quantitative biomarkers capable of objectively tracking muscle preservation. Magnetic resonance imaging particularly fat fraction (FF) and T2 mapping sequences, has emerged as a leading outcome measure for monitoring muscle pathology and treatment response, with MRI parameter changes frequently detectable prior to clinically apparent decline [26-28]. Ultrasound provides complementary information on muscle thickness and echo intensity, while serum creatine kinase (CK) remains a conventional marker of sarcolemmal injury, albeit with limited sensitivity to subtle disease progression [28-29]. Functional assessments including the 6-minute walk test, Medical Research Council sum scores, and quantitative strength measurements via dynamometry should be incorporated to provide comprehensive outcome evaluation [28]. The integration of imaging, biochemical, and functional measures will substantially enhance the sensitivity and specificity of future therapeutic trials [26], [27], [30].

Exercise prescription must be tailored according to muscular dystrophy subtype given the heterogeneity in underlying pathophysiology and exercise tolerance across disease variants. Submaximal aerobic exercise appears appropriate for Duchenne muscular dystrophy, whereas high-resistance training carries potential risk for exacerbating muscle damage and should generally be avoided [16]. Strength training protocols may benefit individuals with facioscapulohumeral and limb-girdle muscular dystrophies, provided they are carefully adapted to minimize excessive fatigue and optimize adherence [2, 3, 14, 15]. Future investigations should refine these subtype-specific recommendations and systematically evaluate their effects on quality of life and psychosocial outcomes [14].

The majority of CBD studies in muscular dystrophy have been conducted in preclinical models or employed non-standardized formulations of uncertain composition and bioavailability [9], [10]. Clinical trials utilizing pharmaceutical-grade CBD preparations, such as FDA-approved formulations developed for other indications, are needed with rigorous dose-escalation protocols and comprehensive safety monitoring [19]. Dose-finding studies represent a critical priority given preclinical evidence of dose-dependent therapeutic and toxic effects [29]. Such trials should additionally examine potential interactions between CBD and exercise interventions as well as other concurrent therapies.

In summary, advancing evidence-based approaches to muscle preservation in muscular dystrophy will require comparative RCTs incorporating validated biomarkers, individualized exercise protocols informed by disease subtype, and carefully controlled investigations of pharmaceutical-grade CBD dosing and safety. These research priorities offer the most promising path toward clarifying the respective and potentially complementary roles of exercise and cannabinoid-based interventions in this challenging clinical population.

## Conclusion

Exercise therapy remains the most substantiated non-pharmacologic intervention for muscle preservation in muscular dystrophy, supported by a growing body of human clinical evidence demonstrating improvements in functional capacity, endurance, and quality of life with acceptable safety when programs are appropriately individualized. In contrast, cannabidiol supplementation represents a promising but experimental strategy, with encouraging preclinical data suggesting anti-inflammatory, anti-fibrotic, and myoprotective effects, yet an absence of adequately powered human trials limits its clinical applicability at present. Although both interventions share convergent mechanisms related to inflammation reduction and oxidative stress modulation, their therapeutic profiles and levels of evidentiary maturity differ substantially. The potential for synergistic benefit through combined exercise and CBD interventions is theoretically compelling but remains unproven and should be approached cautiously. Advancing evidence-based care for muscular dystrophy will require rigorously designed randomized controlled trials incorporating standardized biomarkers, disease subtype–specific protocols, and careful evaluation of safety and dosing, particularly for cannabinoid-based therapies. Until such data are available, exercise therapy should remain the foundation of muscle preservation strategies in MD, while CBD supplementation should be confined to the research setting.

## Key points

- Muscular dystrophy is characterized by progressive muscle degeneration driven by inflammation, oxidative stress, and impaired regeneration.
- Exercise therapy is the most established non-pharmacologic intervention for muscle preservation in MD.
- Aerobic, resistance, and functional exercise programs provide modest improvements in endurance and functional performance.
- Gains in absolute muscle mass and strength with exercise are generally limited and variable across MD subtypes.
- Cannabidiol exhibits anti-inflammatory, anti-fibrotic, and myoprotective effects in dystrophic animal models.
- No adequately powered clinical trials have evaluated CBD supplementation in muscular dystrophy patients.
- Exercise and CBD share overlapping anti-inflammatory and antioxidant mechanisms, suggesting possible synergy.
- Safety, dosing standardization, and long-term effects of CBD in neuromuscular populations remain uncertain.
- Future research should focus on comparative randomized trials, standardized biomarkers, and combination therapy strategies.
